# Investigation of Anisotropic Subsequent Yield Behavior for 45 Steel by the Distortional Yield Surface Constitutive Model

**DOI:** 10.3390/ma13051196

**Published:** 2020-03-06

**Authors:** Yanjun Chang, Zheng Kuang, Runsen Tang, Jianyun Chen, Qiao Song

**Affiliations:** College of Civil Engineering and Architecture, Key Laboratory of Disaster Prevention and Structural Safety of Ministry of Education, Guangxi Key Laboratory of Disaster Prevention and Engineering Safety, Guangxi University, Nanning 530004, China; changyj@gxu.edu.cn (Y.C.); jianyun_chen@126.com (J.C.)

**Keywords:** anisotropic plasticity, subsequent yield behavior, distortional yield surface, constitutive model, 45 steel

## Abstract

The subsequent anisotropic yield behavior of 45 steel was predicted by the distortional yield surface constitutive model, which can describe the anisotropic subsequent yield and the cross effect of metal associating with loading history. The yield characteristics and plastic hardening behaviors of the 45 steel were simulated under three preloading paths including pre-torsion, pre-tension, and pre-tension–torsion. Based on the comparison between the experimental yield stresses and the simulation by the classical Chaboche model, the proposed model can describe the remarkable anisotropic yield behavior related to the loading history, which can effectively describe the sharp point of yield surface in pre-loading direction and the smaller curvature near its opposite direction. It was successfully simulated by the constitutive model proposed that the subsequent distortional yield surface defined by small offset strain and the degradation process of the distortion feature defined by large offset strain.

## 1. Introduction

In the classical plastic theory, isotropic and kinematic hardening are usually used to track the expansion and movement of yield surface, respectively. Kinematic hardening can exhibit certain anisotropic characteristics under the plastic deformation process, in which the position of the subsequent yield surface changes while the size of the subsequent yield surface remains unchanged. To characterize the kinematic hardening behavior, the linear kinematic hardening model was proposed firstly [1,2], and the nonlinear kinematic hardening model was developed by introducing a dynamic recovery term [3]. Chaboche put forward a more general cyclic plasticity macroscopic constitutive based on A-F hardening model proposed by Armstrong and Frederick, which is suitable to characterize the cyclic hardening and softening behavior when the materials are subject to the proportional loading [4,5].

According to the above plastic theory, the initial yield curve and the subsequent yield curve are circles on the $\sigma -\sqrt{3}\tau$ stress plane. However, many experiments have shown the distortion of yield curves, on which the sharp corner along the preloading direction and the smaller curvature near the opposite direction [6,7]. The distortion shape of the subsequent yield surface varies for different metals. Based on the experimental investigation on the yield behavior of steel, copper, and aluminum under proportional tension–torsion preloading, it has shown that the offset strain has an important effect on the shape of the yield surface [6,7,8]. The subsequent yield curve determined by the small offset strain appears as the obvious ‘sharp corner’, and the distortion of the yield curve gradually weakens with the increasing offset strain. However, the distortion of the yield curve remains unchanged determined by different offset strains for some alloys [9,10,11]. Therefore, the classical plastic theory under the Mises theory framework cannot describe the anisotropic subsequent yield surfaces.

To catch the distortion of yield surfaces by the macroscopic method, the distortional yield function was defined by using the distortional deviatoric stress tensor based on the mixed hardening yield function [12]. Feigenbaum [13,14] proposed the distortional yield model by introducing the fourth-order anisotropic tensor into the Hill orthogonal yield function. A general cyclic plastic model was established to track the ratcheting behavior under multi-axial loading, in which 25 distortional variables were introduced into the yield function to consider the rotation of principal stress axes [15]. Baodong et al. [16] simplified the anisotropic yield model by introducing some of the distortion factors into the classical yield function. Jianyun et al. [17] developed an anisotropic constitutive model for distortional yield surface by adding an anisotropic distortional term to the isotropic hardening stress.

The present work focuses on the validation of the new anisotropic constitutive model for 45 steel with obvious yield phenomenon that the stress does not increase (or fluctuate within a small range) when it reaches a certain value, but the strain increases rapidly during initial loading. The model parameters of the anisotropic plastic constitutive model were calibrated by the cyclic tension–compression test and tension test. The experimental yield surfaces under pre-deformation were simulated by the anisotropic yield model. The reasonability of the anisotropic model was demonstrated by comparison and analysis with respect to the simulation results of the Chaboche cyclic plastic model and experimental data.

## 2. Theoretical Model

### 2.1. The Distortional Yield Function

The yield function is the foundation of the plastic constitutive model, as well as the plastic hardening and flow theory. A suitable yield function should be adopted to fit the different plastic behaviors of the diversified materials. The expansion and translation of the subsequent yield surface were modeled by the isotropic and the kinematic hardening in the classical Mises yield theory. Adding the anisotropic distortional function H to the isotropic hardening term, the expression of the anisotropic yield function used in this presentation is:

(1) $$F_{y}=\left\|s-\alpha \right\|-\left(R+\sigma_{0}\right)H\left(w,d\right)$$

where s,α are the deviatoric stress and the back stress and $R,\sigma_{0}$ are the isotropic hardening term and the initial yield stress without any plastic deformation history. H is the distortional function with the variables of the anisotropic distortional factor d and the anisotropic weakening factor w.

According to the Chaboche model [4,5], decomposing the back stress into a multinomial form, the combined nonlinear kinematic laws can be described more accurately. The general form of back stress adopted is:

(2) $$\alpha =\sum_{i=1}^{M}\alpha_{i},{\dot{\alpha }}_{i}=C_{i}\left(\frac{2}{3}a_{i}{\dot{\varepsilon }}^{p}-\alpha_{i}\dot{p}\right)$$

where the back stress includes the linear rule and the momentary strain memory, which decreases gradually with the increase of cumulative plastic strain. M represents the number of terms of back stress. Considering the simplicity and description ability of the model, M=2 is taken here. $a_{i},C_{i}$ are two parameters that characterize the evolution of back stress. The equivalent plastic strain rate $\dot{p}$ of the rate-dependent model is defined by:

(3) $$\dot{p}={\langle \frac{F_{y}}{K}\rangle }^{m}$$

where < > is the MacCauley bracket and K,m are the viscous parameters. $F_{y}$ is the yield function.

The plastic strain rate ${\dot{\varepsilon }}^{p}$ of Equation (2) was deduced with Equation (3) as:

(4) $${\dot{\varepsilon }}^{p}=\sqrt{\frac{3}{2}}{\langle \frac{F_{y}}{K}\rangle }^{m}n$$

where n is the plastic flow tensor and $\dot{p}$ is the equivalent plastic strain rate and defined by:

(5) $$\dot{p}=\sqrt{\frac{2}{3}{\dot{\varepsilon }}^{p}:{\dot{\varepsilon }}^{p}}$$

The isotropic hardening term R in the yield function above is defined as:

(6) R=Q[1−exp(−b⋅p)]

where Q and b are the saturation parameters for isotropic hardening that can be calibrated by cyclic test, and p is the equivalent plastic strain accumulated. For the initial yield surface with zero plastic strain, the following formula can be deduced:

(7) $$a_{i}\left(0\right)=0,\;\;p\left(0\right)=0,\;\;R\left(0\right)=0$$

### 2.2. The Anisotropic Distortional Function

The anisotropic distortional function H in Equation (1) of the anisotropic yield function is given as:

(8) $$H\left(d,w\right)=\left\{ \begin{array}{l} d+\left(1-d\right)\frac{\left\|\alpha -\alpha_{unloading}\right\|}{w\cdot \left\|\alpha_{unloading}\right\|},\;\;\;\;\;\;Preloading,\;Unloading,Reloading\;when\;\frac{\left\|\alpha -\alpha_{unloading}\right\|}{w\cdot \left\|\alpha_{unloading}\right\|}\leq 1\\ 1,\;\;\;\;\;\;\;\;\;\;\;\;\;\;\;\;\;\;\;\;\;\;\;\;\;\;\;\;\;\;\;\;\;\;\;\;\;\;Reloading\;when\;\frac{\left\|\alpha -\alpha_{unloading}\right\|}{w\cdot \left\|\alpha_{unloading}\right\|}\geq 1 \end{array}\right.$$

where α is the back stress which evolves with the loading, $\alpha_{unloading}$ is the back stress of the unloading point, and w is the anisotropic weakening factor calibrated by the experimental subsequent yield surfaces with different offset strains.

The second-order tensor $\alpha_{unloading}$ is equal to the back stress tensor α during the preloading stage and remains unchanged during the unloading process, as follows:

(9) $$\alpha_{unloading}=\alpha ,\;\;\;\;\;\;\;\;\;\;\;\;\;\;Preloading\;and\;Unloading$$

The degradation process of the distortional yield surface from anisotropy to quasi-isotropy can be described with the anisotropic weakening factor. The distortion of the yield surface weakens rapidly when w is relatively small. The back stress evolves during reloading process, and the anisotropic distortional function H changes gradually from d to 1. The anisotropic distortional yield surface will evolve into a yield surface without distortion when the back stress satisfies the condition $\left\|\alpha -\alpha_{unloading}\right\|=w\cdot \left\|\alpha_{unloading}\right\|$ corresponding to H=1.

### 2.3. The Anisotropic Distortional Factor d

Considering the cross effect of the subsequent yield surfaces by test and mesoscopic simulation [18,19,20], the anisotropic distortional factor d for the distortional yield surface is given by:

(10) $$d=\left[1-\beta_{1}+\beta_{1}{\left(n_{1}:n_{2}\right)}^{3}\right]\left[1+\beta_{2}-\beta_{2}{\left(n_{1}:n_{2}\right)}^{2}\right]$$

where $\beta_{1},\beta_{2}$ are the cross effect parameters and the two tensors $n_{1},n_{2}$ are defined as:

(11) $$n_{1}=\frac{s-\alpha }{\left\|s-\alpha \right\|},\;\;n_{2}=\frac{\alpha }{\left\|\alpha \right\|}$$

The cross effect parameters can be calibrated by the anisotropic yield with tension or torsion experiment (cf. Section 3.1). The distortion of yield surface disappears when the cross effect parameters are equal to zero, $\beta_{1}=\beta_{2}=0$.

### 2.4. The Flow Directions of the Anisotropic Model

According to the orthogonal flow law, the gradient components of the anisotropic yield function can be deduced with the partial derivative of the yield function:

(12) $$\frac{\partial F_{y}}{\partial \sigma }=\frac{s-\alpha }{\left\|s-\alpha \right\|}-\left(\sigma_{0}+R\right)\frac{\partial H}{\partial \sigma }.$$

In the stage of reloading, combined with Equations (8), (11), and (12), the gradient components can be written as:

(13) $$\frac{\partial F_{y}}{\partial \sigma }=n_{1}-\left(\sigma_{0}+R\right)\left(1-\frac{\left\|\alpha -\alpha_{unloading}\right\|}{w\cdot \left\|\alpha_{unloading}\right\|}\right)\frac{\partial d}{\partial \sigma }$$

According to Equation (9), in the case of preloading and unloading, H=1, $\frac{\partial H}{\partial \sigma }=0$, where the plastic flow direction is perpendicular to the cylindrical yield surface, $\frac{\partial F_{y}}{\partial \sigma }=n_{1}$. In the case of the preload phase and $\left\|\alpha -\alpha_{unloading}\right\|\geq w\cdot \left\|\alpha_{unloading}\right\|$, $\frac{\partial H}{\partial \sigma }=0$ and $\frac{\partial F_{y}}{\partial \sigma }=n_{1}$.

The term in Equation (13), $\frac{\partial d}{\partial \sigma }$, can be calculated according to the expression of the anisotropic distortional factor d as:

(14) $$\frac{\partial d}{\partial \sigma }=\left[-2\beta_{2}\left(1-\beta_{1}\right)\left(n_{1}:n_{2}\right)+3\beta_{1}\left(1+\beta_{2}\right){\left(n_{1}:n_{2}\right)}^{2}-5\beta_{1}\beta_{2}{\left(n_{1}:n_{2}\right)}^{4}\right]\frac{\partial \left(n_{1}:n_{2}\right)}{\partial \left(s-\alpha \right)}.$$

Due to the back stress is irrelevant to s−α, the partial derivative of the tensor $n_{2}$ is zero, as:

(15) $$\frac{\partial n_{2}}{\partial \left(s-\alpha \right)}=0$$

Therefore,

(16) $$\frac{\partial \left(n_{1}:n_{2}\right)}{\partial \left(s-\alpha \right)}=\frac{\partial n_{1}}{\partial \left(s-\alpha \right)}:n_{2}=\frac{I-n_{1}⊗n_{1}}{\left\|s-\alpha \right\|}:n_{2}$$

To simplify the expression above, the parameter $\beta_{3}$ and a fourth-order tensor J are defined as follows:

(17) $$J=\frac{I-n_{1}⊗n_{1}}{\left\|s-\alpha \right\|}$$

(18) $$\beta_{3}=-2\beta_{2}\left(1-\beta_{1}\right)\left(n_{1}:n_{2}\right)+3\beta_{1}\left(1+\beta_{2}\right){\left(n_{1}:n_{2}\right)}^{2}-5\beta_{1}\beta_{2}{\left(n_{1}:n_{2}\right)}^{4}$$

Combining with Equations (1) and (13)–(18), the norm for the gradient of the anisotropic yield surface can be calculated as:

(19) $$\left\|\frac{\partial F_{y}}{\partial \sigma }\right\|==\left[n_{1}-\left(\sigma_{0}+R\right)\beta_{3}J:n_{2}\right]:\left[n_{1}-\left(\sigma_{0}+R\right)\beta_{3}J:n_{2}\right]=1+\beta_{3}{}^{{}^{2}}\frac{1-{\left(n_{1}:n_{2}\right)}^{2}}{d^{2}}$$

(20) $$\frac{1}{G}=1+\beta_{3}{}^{2}\frac{1-{\left(n_{1}:n_{2}\right)}^{2}}{d^{2}}$$

The plastic flow unit tensor n can be unitized with the gradient components of the anisotropic yield surface

(21) n=∂Fy∂σ‖∂Fy∂σ‖=G[n1−(σ0+R)β3J:n2]

Integrating Equations (9), (13), and (21), the plastic flow tensor n on different loading stages can be summarized as:

(22) $$n=\left\{ \begin{array}{ll} G\left[n_{1}-\left(\sigma_{0}+R\right)\beta_{3}J:n_{2}\right], & Preloading,\;Unloading,Reloading\;when\;\;\frac{\left\|\alpha -\alpha_{unloading}\right\|}{w\cdot \left\|\alpha_{unloading}\right\|}\leq 1\\ n_{1} & Reloading\;and\;\frac{\left\|\alpha -\alpha_{unloading}\right\|}{w\cdot \left\|\alpha_{unloading}\right\|}\geq 1 \end{array}\right.$$

where the stress tensors $n_{1}$ and $n_{2}$ are normalized by the back stress tensor α and the stress tensor s−α as Equation (11).

Substituting the plastic flow tensor n into Equation (4), the total plastic strain $\varepsilon^{p}$ can be accumulated. According to the strain decomposition law, the relationship between stress and strain is expressed with the fourth-order stiffness tensor D, as:

(23) $$\sigma =D:\varepsilon^{e}=D:\left(\varepsilon -\varepsilon^{p}\right)$$

The effect of loading history on the plastic behavior can be considered by the anisotropic constitutive model.

## 3. Calibration of Model Parameters and FEM Model

### 3.1. Calibration of Model

#### 3.1.1. Calibration of Anisotropic Parameters

Because $n_{1}$ and $n_{2}$ are the second-order unit tensors, the angle θ can be defined as:

(24) $$\theta =arccos\left(n_{1}:n_{2}\right).$$

Substituting Equation (24) into Equation (10), the anisotropic distortional factor d is rewritten as:

(25) $$d=\left[1-\beta_{1}+\beta_{1}{\left(cos\theta \right)}^{3}\right]\left[1+\beta_{2}{\left(sin\theta \right)}^{2}\right].$$

For tension–torsion stress complex loading, the angle between the preloading and the reloading direction in the $\sigma -\sqrt{3}\tau$ stress plane is equal to the angle θ given in deviatoric stress space [21]. According to the strain decomposition within the assumption of small strain, the parameters $\beta_{1},\beta_{2}$ can be calibrated by the distortion yield surface and setting the angle with $\theta =0^{\circ }$ and $\theta =90^{\circ }$, which correspond to the proper angle the preloading direction and the reloading direction. Therefore, the parameters $\beta_{1},\beta_{2}$ are so-called as the cross effect parameters because of their dominant effect on the distortion yield surface.

**Figure 1:**
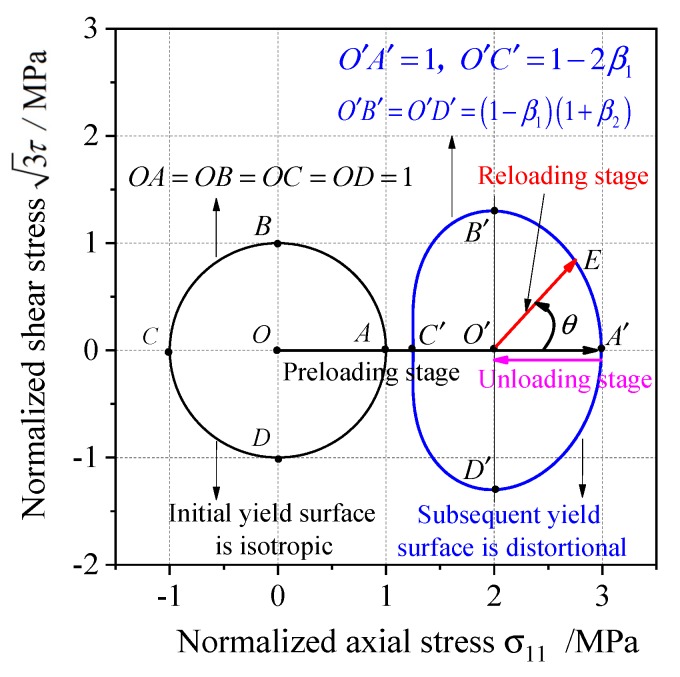
Schematic of anisotropic distortion subsequent yield curve in the $\sigma -\sqrt{3}\tau$ stress plane.

To measure the yield stresses of the yield curve, the three loading stages including the preloading, unloading, and reloading processes are essential. It is supposed that the initial yield surface is isotropic according to the experimental result for 45 steel, so the initial yield curve is a circle in $\sigma -\sqrt{3}\tau$ stress plane (the solid black line in Figure 1). The pretension loading, unloading, and tension–torsion complex reloading tests that the loading path is $O\to A^{\prime }\to O^{\prime }\to E$ in $\sigma -\sqrt{3}\tau$ stress plane can be used to calibrate the anisotropic parameters. The point $O^{\prime }$ in Figure 1 corresponds to the back stress $\alpha^{*}$ during the unloading stage, and the tension–torsion complex reloading path is $\sigma -\alpha^{*}$ which corresponds to the ray line $\vec{O^{\prime }E}$ in $\sigma -\sqrt{3}\tau$ stress plane (the solid red line in Figure 1). The yield stresses along different reloading paths can be obtained by changing the angle $\theta =∠A^{\prime }O^{\prime }E$ shown in Figure 1, which link together the whole yield curve in the $\sigma -\sqrt{3}\tau$ stress plane.

For the subsequent yield surface, $A^{\prime }B^{\prime }C^{\prime }D^{\prime }$ is symmetric to $A^{\prime }C^{\prime }$ in Figure 1, and the length of line segments can be calculated by Equation (25) with $\theta =90^{\circ }$ as $O^{\prime }B^{\prime }=O^{\prime }D^{\prime }=\left(1-\beta_{1}\right)\left(1+\beta_{2}\right)$. Similarly, the length of line segments of the distortional yield curve can be calculated by Equation (25) with $\theta =0^{\circ }$ as $O^{\prime }A^{\prime }=1$, $O^{\prime }C^{\prime }=1-2\beta_{1}$. The anisotropic model parameters $\beta_{1}$, $\beta_{2}$ can be calculated by the line segments of the subsequent yield curve in Figure 1:

(26) $$\beta_{1}=\frac{O^{\prime }A^{\prime }-O^{\prime }C^{\prime }}{2\cdot O^{\prime }A^{\prime }},\;\beta_{2}=\frac{B^{\prime }D^{\prime }}{A^{\prime }C^{\prime }}-1$$

The distortion along the preloading stress axis is dominated by the cross effect parameter $\beta_{1}$, and the distortion vertical to the preloading stress axis is controlled by the cross effect parameters $\beta_{1}$,$\beta_{2}$ together. The distortional feature that sharp in preloading direction and the lower curvature near the reverse direction become more obvious with the higher value of the cross effect parameter $\beta_{1}$. The yield along the direction vertical to the preloading path occurs more easily with lower a value than the cross effect parameter $\beta_{2}$. The distortion of the subsequent yield surface vanishes when the cross effect parameters are zero, $\beta_{1}=\beta_{2}=0$, and the subsequent yield surface becomes isotropic.

Metals gradually harden and the distortion of the subsequent yield surface gradually weakens when the offset strain increases [20]. The anisotropic degradation factor w describes the evolution process of the distortional yield surface and can be calibrated by a few yield curves corresponding to different offset strains in tension–torsion stress plane. The higher the value of the anisotropic degradation factor w, the slower degradation process from the anisotropic distortional yield surface to the quasi-isotropic yield surface. The anisotropic degradation factor w usually varies for different metals, and it is determined with 45 steel in this presentation.

In general, the cross effect parameters $\beta_{1}$, $\beta_{2}$ and the degradation factor w can be calibrated by the distortion yield surface characteristics under tension–torsion biaxial loading, which are not included in the Chaboche model.

#### 3.1.2. Calibration of Hardening Parameters

**Figure 2:**
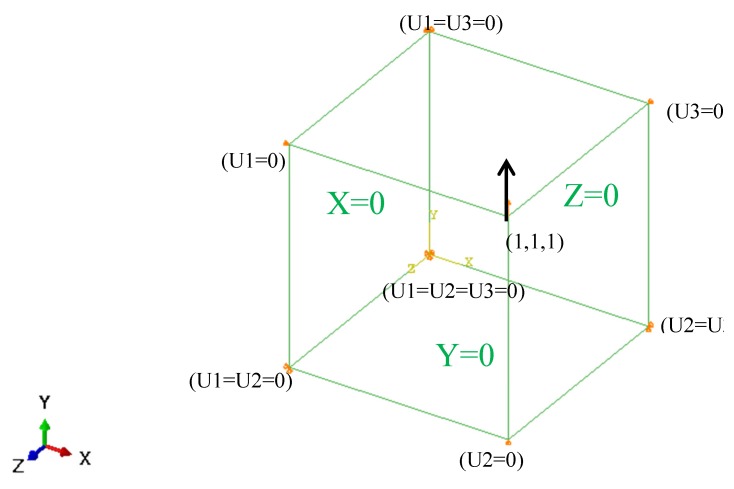
Loading method and the boundary conditions on the representative element.

**Figure 3:**
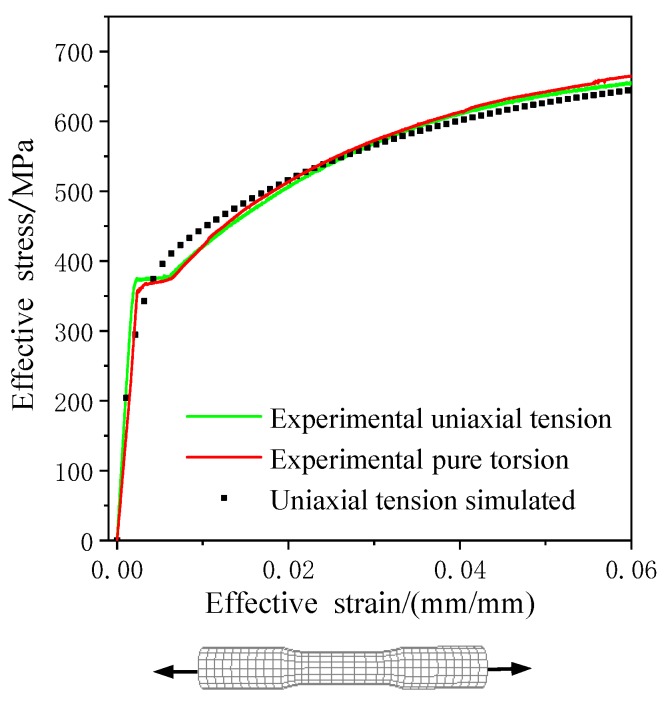
The experimental equivalent stress–strain curves of 45 steel under tensile and torsion loadings [22] and tensile simulation by the anisotropic model.

**Figure 4:**
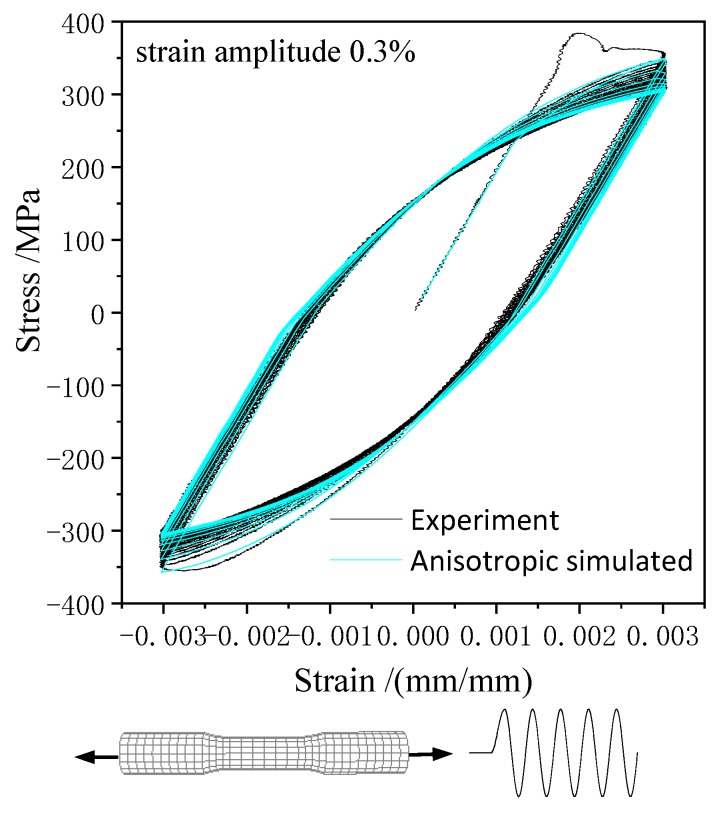
Uniaxial tension–compression simulation by the anisotropic model versus the experimental hysteresis loop [22].

Based on the ABAQUS simulation platform, the user-defined material subroutine (UMAT) of the presented anisotropic constitutive model was applied to study the elastoplastic behavior of 45 steel. To improve the accuracy and efficiency of the calibration, a single three-dimensional eight-node reduced integration element (C3D8R) element was employed, as shown in Figure 2. The deformation of the element is consistent and keeps the six surfaces planar by giving the specific displacement boundary condition. The parameters of anisotropic hardening model were calibrated by the cyclic tension–compression test and uniaxial tension test, which the axial loading applied along *y*-axis at reference point X = Y = Z = 1, as shown in Figure 2. Therefore, the different stress–strain response with various hardening parameters of the anisotropic plastic model can be estimated under monotonic tensile loading and cyclic tension–compression loading, as shown in Figure 3 and Figure 4.

The hardening parameters of the anisotropic plastic model including isotropic hardening parameters $b,\;Q,\;\sigma_{0},\;K,\;m$, linear kinematic hardening parameters $a_{1},\;a_{2}$, and nonlinear kinematic hardening parameters $C_{1},\;C_{2}$. By fitting the stress–strain curve simulated to the experimental data under axial tensile loading and tension–compression cyclic loading, the parameters of the anisotropic plastic model for 45 steel were determined as shown in Table 1. It was shown that the mechanical behavior of 45 steel simulated by the anisotropic constitutive model under axial tensile and cyclic loading is highly consistent with the experimental results.

### 3.2. Finite Element Model

**Figure 5:**
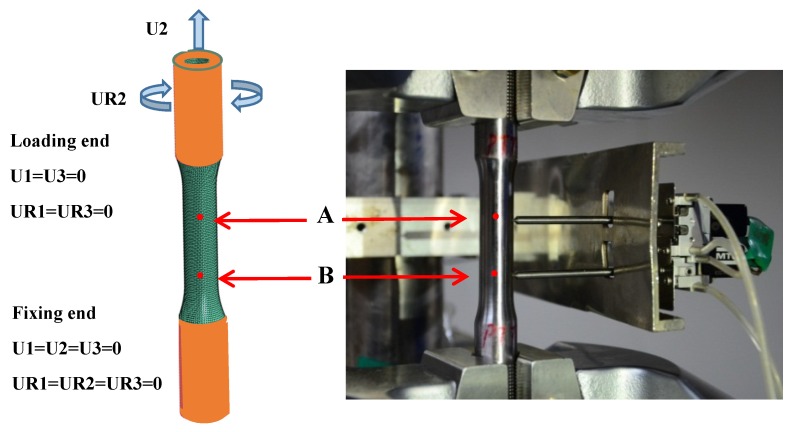
The thin-walled FEM model under the combined tension–torsion loading.

Thin-walled tubular specimen for the yield surface tests was employed such that the gauge length was 50 mm, the outer and inner diameters were 12.5 mm and 10 mm. A full-scale FEM model was adopted to ensure the identical geometric and mechanical boundary with the experiment, which included 25,584 C3D8R elements and 32,242 nodes. The loading and boundary conditions in the FEM model were performed by coupling the elements of the loading end and fixing end (the orange region in Figure 5) with the reference points, respectively, as shown in Figure 5. All six degrees of freedom were set as zero at the fixed end. The combined tension–torsion loadings were performed on the second axial and the fifth rotational degrees of freedom, and the remaining four degrees of freedom were set to zero U1=U3=UR1=UR3=0. The corresponding user-defined material (UMAT) of ABAQUS software was applied to obtain the yield behavior under the combined tension–torsion loading.

The stress distribution of the thin-walled pipe is uniform with respect to tension and torsion loadings. The displacements of the two key nodes A and B were acquired corresponding to the displacement of the extensometer fixed on the real specimen, as shown in Figure 5. The axial and torsional displacements of the two points were recorded, and the axial force and torque on the loading end were output. Therefore, the tensile and shear stresses and strains can be calculated as follows:

(27) $$\sigma =\frac{4F}{\pi (D^{2}-d^{2})},\tau =\frac{16TD}{\pi (D^{4}-d^{4})}$$

(28) $$\varepsilon =\frac{U2^{A}-U2^{B}}{AB},\gamma =\frac{\left(UR2^{A}-UR2^{B}\right)\left(D+d\right)}{4AB}$$

where *F* and *T* are the axial force and torque, respectively, D and d are the outer and internal diameters at the gauge segment. $U2_{}^{A},\;UR2_{}^{A}$ are the axial and torsional displacements of points *A*, and $U2_{}^{B},\;UR2_{}^{B}$ are the axial and torsional displacements of points *B*, as shown in Figure 5. The length of line segment AB is the same as that of the extensometer.

Using the expressions of Equation (28), the equivalent stress and strain under the tension–torsion loading can be defined as:

(29) $$\sigma_{eq}=\sqrt{\sigma^{2}+3\tau^{2}},\varepsilon_{eq}=\sqrt{\varepsilon^{2}+\frac{\gamma^{2}}{3}}$$

## 4. Determination Method for the Subsequent Yield Surfaces

To investigate the subsequent yield behavior of the materials, the multi-sample method is more proper than the single-sample method [19]. Therefore, the multi-sample method was used to determine the yield stresses along every loading paths in this paper. The simulated yield stresses were measured by the offset strain method which is the same as the experimental tests of 45 steel [20].

**Figure 6:**
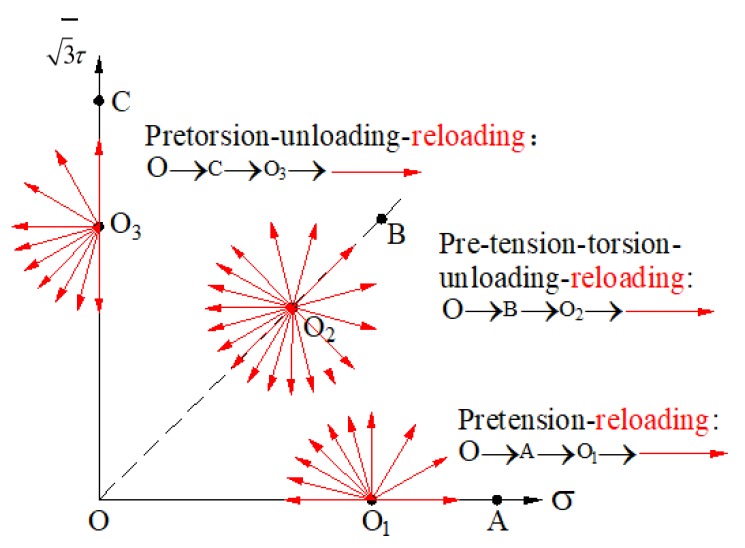
Schematic of stress paths for subsequent yield curves with pretension, pre-torsion, and pre-tension–torsion loading.

To verify the wide applicability of the anisotropic distortional yield surface constitutive model for 45 steel, the subsequent yield surfaces of 45 steel under three typical preloading conditions including pre-tension, pre-torsion, and pre-tension–torsion proportional loading were predicted with this model. The relatively smaller unloading process was performed to avoid the plastic deformation, and the equivalent stresses at the unloading point were 384.7 MPa for 3% preloading equivalent strain and 276.8 MPa for 0.5% preloading equivalent strain, respectively. Under pre-tension and pre-torsion preloading conditions, the reloading processes were along the loading angles of 0, 30, 60, 90, 105, 120, 135, 150, 165, and 180 in the $\sigma -\sqrt{3}\tau$ stress plane, and another half yield surface was obtained by a symmetric method based on the decoupling assumption of tension–torsion, as shown in Figure 6. The reloading angles for yield surface include 0, 30, 60, 90, 105, 120, 135, 150, 165, 180, −165, −150, −135, −120, −105, −90, −60, and −30 in the $\sigma -\sqrt{3}\tau$ stress plane under pre-tension–torsion proportional loading. The number of reloading paths is greater than that of real experiment tests for smoother yield surfaces of 45 steel [20].

## 5. The Simulation Results for the Yield Surfaces

**Figure 7:**
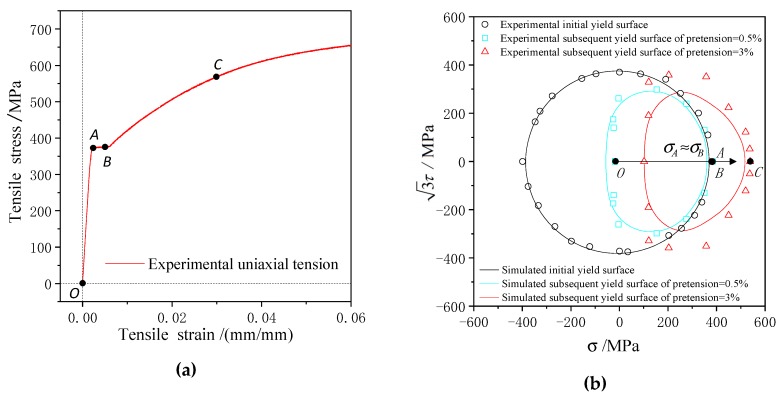
(**a**) The tensile stress–strain curve of 45 steel; (**b**) the initial yield curve and the subsequent yield curves simulated versus the experimental results [22] under pretension strains of 0.5% and 3%.

There is an obvious yield stage on the tensile stress–strain curve of 45 steel, in which the tensile total strain covers approximately 0.2%–0.6%. The yield surface reduces to the yield curve in the $\sigma -\sqrt{3}\tau$ stress plane and the subsequent yield surfaces were simulated under two pretension strain conditions, 0.5% and 3%, as shown in Figure 7. The initial yield curve with the offset strain of $2\times 10^{-4}$ is nearly circular including initial yield point A in Figure 7a,b under monotonic tensile loading.

The hardening process takes place and the tensile stress ascends obviously when the pretension strain increases from 0.5% (point B in Figure 7a) to 3% (point C in Figure 7a) [22]. Therefore, the subsequent yield surface moves toward larger tensile stress with increasing pretension strain. The subsequent yield curves (cf. the blue and red lines in Figure 7b) are smaller than the initial yield curve (cf. the black circle in Figure 7b). The subsequent yield curves simulated of 45 steel under two pretension loadings exhibits significant smooth anisotropy, as shown in Figure 6, which is characterized by a sharp region near the direction of preloading and the lower curvature on the opposite direction. The yield curves simulated by the anisotropic yield model (cf. the blue, red, and black curves in Figure 7a) agree well with the experimental yield results [22], and it was affirmed that this anisotropic yield model can reflect the distortional subsequent yield behavior of 45 steel.

**Figure 8:**
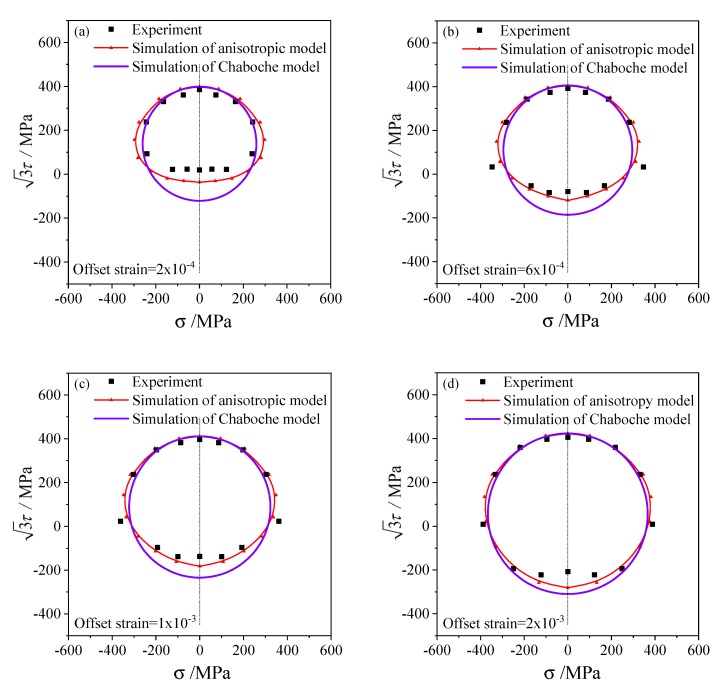
The subsequent yield curves simulated and the experimental results on the $\sigma -\sqrt{3}\tau$ stress plane with pre-torsion of equivalent strain 0.5% and unloading to the equivalent stress of 236.3 MPa: (**a**) the offset strain is 2 × 10^−4^; (**b**) the offset strain is 6 × 10^−4^; (**c**) the offset strain is 1 × 10^−3^; (**d**) the offset strain is 2 × 10^−3^.

**Figure 9:**
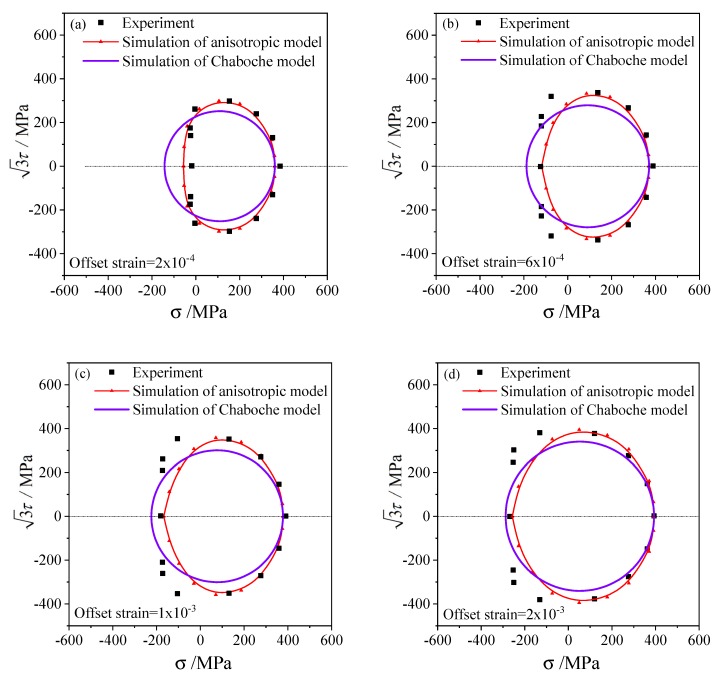
The subsequent yield curves simulated and the experimental results on the $\sigma -\sqrt{3}\tau$ stress plane with pretension strain 0.5% and unloading to the equivalent stress of 276.8 MPa: (**a**) the offset strain is 2 × 10^−4^; (**b**) the offset strain is 6 × 10^−4^; (**c**) the offset strain is 1 × 10^−3^; (**d**) the offset strain is 2 × 10^−3^.

**Figure 10:**
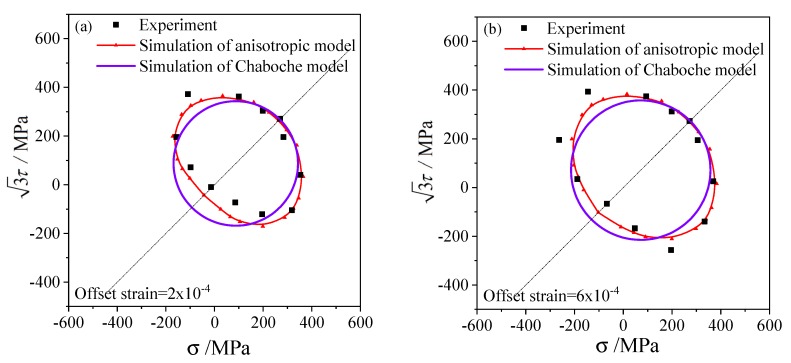

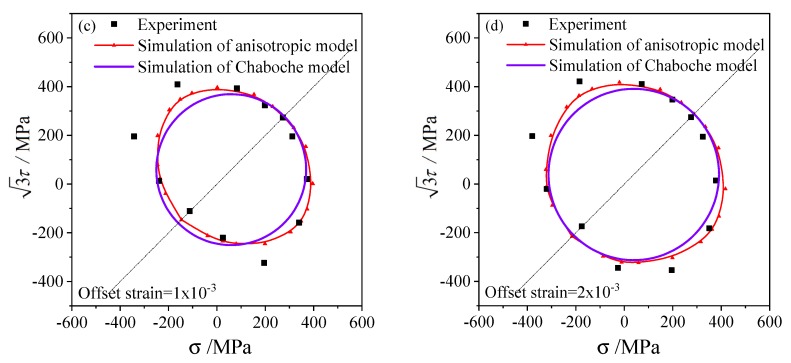
The subsequent yield curves simulated and the experimental results on the $\sigma -\sqrt{3}\tau$ stress plane with proportional pre-tension–torsion equivalent strain 0.5% and unloading to the equivalent stress of 198.2 MPa: (**a**) the offset strain is 2 × 10^−4^; (**b**) the offset strain is 6 × 10^−4^; (**c**) the offset strain is 1 × 10^−3^; (**d**) the offset strain is 2 × 10^−3^.

According to the three loading paths and offset strain with 0.5% for the subsequent yield stresses, The subsequent yield surfaces determined under three pre-deformations including torsion, tensile, and tension–torsion were simulated and shown in Figure 8, Figure 9 and Figure 10, respectively. To compare with the experimental results of 45 steel [20], the offset strains of $2\times 10^{-4}$, $6\times 10^{-4}$, $1\times 10^{-3}$ and $2\times 10^{-3}$ were chosen to simulate the subsequent yield behavior. In addition, the results simulated by the anisotropic distortion yield surface model were also compared with the simulation by the classical Chaboche model.

The elastic and hardening parameters used in the anisotropic model are the same as those in the Chaboche model, as presented in Table 1. Since the Chaboche plastic model combines isotropic and kinematic hardening without distortional hardening, the corresponding subsequent yield curves are standard circles in the $\sigma -\sqrt{3}\tau$ stress plane, as shown by the purple lines in Figure 8, Figure 9 and Figure 10. Therefore, the Chaboche model cannot capture the non-circular characteristics from the experiment. The comparison between the results of the Chaboche model and the distortional yield model shows that they can display the expansion and translation of the subsequent yield. Moreover, the anisotropic model can characterize the distortion of subsequent yield and are generally better than those of the Chaboche model.

The anisotropic distortion yield curves expand when the offset strain increases, which are the same as the experimental phenomenon. The cross effect of the anisotropic yield surface is more prominent with a smaller offset strain, in which the higher curvature grows along the preloading direction while the lower curvature appears near the reverse direction. As the offset strain increases from $2\times 10^{-4}$ to $2\times 10^{-3}$, the cross effect of the distortional yield curves gradually weakens and the anisotropic yield curves gradually approach the isotropic circular which is consistent with the experimental results.

Under small offset strains such as $2\times 10^{-4}$ and $6\times 10^{-4}$ (Figure 8a,b, Figure 9a,b, Figure 10a,b), the yield stresses calculated by this model can fit the experimental points well. The anisotropic model can successfully capture the distortional characteristics of the experimental subsequent yield curves for higher offset strains such as $1\times 10^{-3}$ and $2\times 10^{-3}$(Figure 8c,d, Figure 9c,d and Figure 10c,d).

To accurately evaluate the effectiveness of the distortional constitutive model, the errors $Err_{aniso}$ and $Err_{Chabo}$ between the yield surfaces simulated by the anisotropic model and the Chaboche model and the experimental results under different preloading conditions and offset strains were calculated by:

(30) $$Err_{aniso}{}^{ij}=\frac{1}{N}\sum_{k=1}^{N}\frac{{\left\|\sigma_{aniso}^{ij}-\sigma_{test}^{ij}\right\|}_{k}}{{\left\|\sigma_{test}^{ij}-\sigma_{unloading}^{ij}\right\|}_{k}},Err_{Chabo}{}^{ij}=\frac{1}{N}\sum_{k=1}^{N}\frac{{\left\|\sigma_{Chabo}^{ij}-\sigma_{test}^{ij}\right\|}_{k}}{{\left\|\sigma_{test}^{ij}-\sigma_{unloading}^{ij}\right\|}_{k}}$$

where the $Err_{aniso}{}^{ij}$ and $Err_{Chabo}{}^{ij}$ represent, respectively, the mean errors of the stresses simulated by the anisotropic model and the Chaboche model with the i-th offset strain and the j-th pre-deformation type, and the k indicates the k-th reloading direction, and the N is the total number of reloading directions according to the loading paths mentioned in Section 4, and ‖‖ is the two-norm of the stress tensor here. In other words, the mean error $Err_{aniso}{}^{ij}$ was counted along each yield curve in Figure 8, Figure 9 and Figure 10 under one offset strain and one preloading condition given.

The average values of four offset strains and three preloading conditions for the anisotropic model and the Chaboche model can be obtained, respectively, by:

(31) $$\bar{Err_{aniso}}=\frac{1}{12}\sum Err_{aniso}{}^{ij},\bar{Err_{Chabo}}=\frac{1}{12}\sum Err_{Chabo}{}^{ij}\;\;\;\;\;\;\;\;\;\;\left(i=1\sim 4,\;\;\;j=1\sim 3\right)$$

where the $\bar{Err_{aniso}}$ and $\bar{Err_{Chabo}}$ are the average errors of the anisotropic model and the Chaboche model, and the total numbers of i is four and j is three.

The standard deviations of the two models and every yield curves in Figure 8, Figure 9 and Figure 10 were calculated with the errors in Equations (30) and (31). The errors and standard deviations of the yield surface calculated by the anisotropic model and the Chaboche model under every offset strain and preloading conditions were shown in Table 2. The mean error of 9.49% and standard deviation of 6.56% of the anisotropic model were generally smaller than that of the Chaboche model, as shown in the last column of Table 2. It was demonstrated that the prediction accuracy under different offset strain and preloading condition is obviously different. Under the pre-torsion loading, the dispersion of yield stress determined by small offset strain is significant, and it gradually decreases with the increase of offset strain. Most of the errors for simulations by the anisotropic model under every offset strains and preloading conditions are close and range from 6% to 12%.

## 6. Discussions and Conclusions

Compared with the experimental results and the simulations of the Chaboche model, the anisotropic distortional yield surface model proposed in this paper can better describe the distortional yield characteristics of 45 steel after preloading. The prediction accuracy of the anisotropic model is higher than that of the Chaboche model, with an average error of 9.49% and a standard deviation of 6.56%, which has proved the rationality and effectiveness of the anisotropic distortion yield surface model in capturing the anisotropic distortion characteristics. The anisotropic distortional yield surface model can describe the process of the anisotropic yield surface extending to isotropic yield surface under reloading. The results simulated by the anisotropic model under pre-torsion and pre-tension–torsion loadings generally fit better than those under pretension loading except for a few yield points.

Based on the analysis of the subsequent yield curves with different offset strains, the nonlinear hardening process is obviously different along various reloading paths and can be predicted by the degradation of the cross effect distortion along the directions reverse to the preloading direction.

It should be indicated that the better simulation precision of the new anisotropic model attributes to the three additional parameters that are not required by the Chaboche model.

## Figures and Tables

**Table 1:** The parameters of the anisotropic plastic model for 45 steel.

	Elastic Constants		Isotropic Hardening Parameters					Kinematic Hardening Parameters				Anisotropic Hardening Parameters		
	Ε	ν	b	Q	$\sigma_{0}$	K	m	$a_{1}$	$C_{1}$	$a_{2}$	$C_{2}$	$\beta_{1}$	$\beta_{2}$	w
Unit	GPa	-	-	MPa	MPa	MPa	-	MPa	-	MPa	-	-	-	-
anisotropic model	193	0.33	1.05	−25	160	80	200	90	800	210	35	0.3	0.8	2
Chaboche model	193	0.33	1.05	−25	160	80	200	90	800	210	35	0	0	0

**Table 2:** The errors and the standard deviation of the yield curves simulated by the anisotropic model and the Chaboche model.

Error (%) (Standard Deviation) (%)		Offset Strain				Mean (%)
		2 × 10^−4^	6 × 10^−4^	1 × 10^−3^	2 × 10^−3^	
Simulation by the Chaboche model	Pre-tension loading	20.24 (10.20)	14.75 (7.27)	13.18 (4.78)	8.27 (5.99)	14.82 (9.74)
	Pre-torsion loading	25.11 (25.65)	15.21 (10.56)	12.44 (7.59)	8.63 (6.92)	
	Pre-tension–torsion loading	19.15 (13.76)	14.90 (8.17)	13.74 (8.57)	12.20 (7.43)	
Simulation by the anisotropic model	Pre-tension loading	8.07 (6.57)	10.49 (5.77)	9.20 (6.93)	7.04 (6.14)	9.49 (6.56)
	Pre-torsion loading	15.93 (6.81)	7.56 (4.77)	6.40 (4.54)	6.26 (5.20)	
	Pre-tension–torsion loading	12.01 (9.68)	9.38 (7.27)	10.38 (7.58)	11.11 (7.41)	
